# Comparison of macroscopic abnormalities in patients receiving routine pantoprazole when compared to placebo

**DOI:** 10.1186/2197-425X-3-S1-A980

**Published:** 2015-10-01

**Authors:** MJ Summers, SP Selvanderan, MP Plummer, ME Finnis, Y Ali Abdelhamid, MB Anderson, MJ Chapman, CK Rayner, AM Deane

**Affiliations:** Intensive Care Unit, Royal Adelaide Hospital, Adelaide, Australia; Discipline of Acute Care Medicine, The University of Adelaide, Adelaide, Australia; Discipline of Medicine, The University of Adelaide, Adelaide, Australia; Department of Gastroenterology and Hepatology, Royal Adelaide Hospital, Adelaide, Australia

## Introduction

Gastrointestinal (GI) mucosal abnormalities have been observed within 24 h of admission to ICU and are thought to represent a 'stress' response. While acid-suppressive drugs are frequently administered to ICU patients, for stress ulcer prophylaxis, there are sparse data to support its efficacy.

## Objectives

To compare the presence of macroscopic mucosal abnormalities during upper GI endoscopy in mechanically ventilated, critically ill patients randomised to receive pantoprazole or placebo.

## Methods

Ten patients were evaluated (6 M:4 F; age 41.7 ± 4.3 y; admission APACHE II score 19.4 ± 1.9; pantoprazole n = 4, and placebo n = 6) from a larger prospective study, in which 209 mechanically ventilated patients were randomised to receive IV pantoprazole (40 mg) or placebo in a double-blind fashion. Patients who received ≥ 5 doses of pantoprazole/placebo (80 patients in total) were eligible for the endoscopy sub-study. Written and informed consent was obtained from the patient's next of kin. The presence of one or more macroscopic abnormalities (erythema or oedema, erosions, ulcerations and naso-gastric tube lesions) and GI bleeding (petechial or sub-mucosal bleeding, haematoma, coffee-ground material and frank blood) were assessed at endoscopy by a single experienced gastroenterologist who was blinded to the intervention. Data are mean ± SEM or median (IQR) and were compared using Mann-Whitney U tests or chi-squared tests as appropriate.

## Results

Patients had received pantoprazole 8.8 ± 0.3 doses or placebo 10.7 ± 1.1 doses prior to endoscopy (p = 0.21). One patient in the pantoprazole group was also receiving corticosteroids, and another received regular non-steroidal anti-inflammatory drug therapy. Patient's haemoglobin on the day of endoscopy were similar (pantoprazole: 89.8 ± 6.0 vs. placebo: 83.7 ± 3.7g/dL; p = 0.52), one patient (receiving pantoprazole) had received a blood transfusion prior to endoscopy. All patients were receiving enteral nutrition prior to endoscopy. Upper GI mucosal abnormalities were observed in a total of 3 patients treated with pantoprazole (75%) and 4 patients treated with placebo (66%), without a difference between treatments (p = 0.78). Observed abnormalities included

(i) erythema/oedema/erosions (pantoprazole: 2 patients vs. placebo: 2 patients)

(ii) naso-gastric tube-related lesions (3 vs. 2),

(iii) petechial or sub-mucosal bleeding (0 vs. 2), and (iv) duodenal ulcer, (0 vs. 1); the latter was not associated with bleeding but the patient was switched to open label pantoprazole.

## Conclusions

Upper GI mucosal abnormalities may occur frequently in mechanically ventilated patients. However, in this small cohort, the prevalence of mucosal abnormalities does not appear to be substantially reduced by administration of prophylactic pantoprazole.

## Grant Acknowledgment

This project was funded by a Royal Adelaide Hospital Research Foundation project grant.

